# Using *PCD*’s First-Ever External Review to Enhance the Journal’s Worldwide Usefulness to Researchers, Practitioners, and Policy Makers

**DOI:** 10.5888/pcd15.180133

**Published:** 2018-03-29

**Authors:** Leonard Jack

**Figure:**
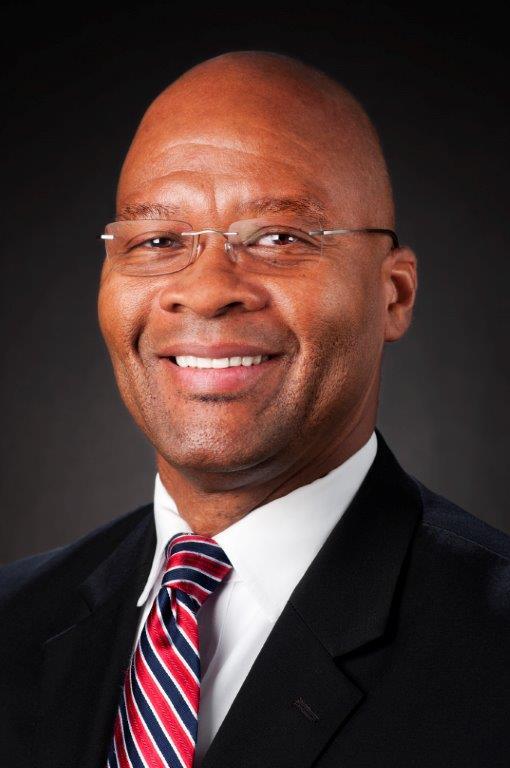
Leonard Jack Jr, PhD, MSc

*Preventing Chronic Disease* (*PCD*) was established in 2004 by the Centers for Disease Control and Prevention (CDC) to enhance the science base on effective public health approaches to prevent and control chronic disease. After 14 years of progress, *PCD* conducted its first-ever external review to identify ways for the journal to continue to enhance its usefulness for its audience of researchers, practitioners, and policy makers. In June 2017, *PCD* invited a panel of 7 nationally recognized experts in scientific publishing (Appendix) to respond to key questions about *PCD*’s mission, quality of scientific content, scope of operation, intended audience, and future direction.

The panel’s overall assessment of *PCD* was that it is well-positioned to continue its trajectory of growth and success. In particular, the panel complimented the journal’s leadership, innovation in scientific publishing, increase in national visibility, and superior customer service provided to its editorial board, associate editors, authors, readers, and peer reviewers. On the basis of the panel’s recommendations and in consultation with the journal’s editorial board, associate editors, and National Center for Chronic Disease Prevention and Health Promotion (NCCDPHP) leadership, I am pleased to share these enhancements to the journal.

## Refining *PCD*’s Vision and Mission Statement

*PCD* was encouraged by the external review panel to refine its vision and mission statement. *PCD* will maintain the journal’s focus on providing current, top-quality content to public health practitioners, researchers, and policy makers. *PCD* vision and mission statements now clearly reflect the journal’s commitment to disseminating respected content worldwide.

**Vision.** *PCD* will serve as an influential journal in the dissemination of proven and promising public health findings, innovations, and practices with editorial content respected for its integrity and relevance to chronic disease prevention.

**Mission statement.** The mission of *PCD* is to promote dialogue among researchers, practitioners, and policy makers worldwide on the integration and application of research findings and practical experience to improve population health.

## Focusing on Topics Areas of Greatest Interest

*PCD* has been in existence long enough to better focus the primary topic areas of greatest interest to the journal. *PCD* has refined its primary focus to 4 main areas:

- Development, implementation, and evaluation of population-based interventions to prevent chronic diseases and control their effect on quality of life, illness, and death.
- Behavioral, psychological, genetic, environmental, biological, and social factors that influence health.
- Interventions that reduce the disproportionate incidence of chronic diseases among at-risk populations.
- Development, implementation, and evaluation of public health law and health-policy–driven interventions.

These 4 areas of interest represent areas in which the journal now seeks to expand its content. On its About the Journal webpage, *PCD* will provide examples of the types of articles and content of greatest interest to the journal under each of these topic areas. Submissions to *PCD* that merely describe programs, theoretical frameworks, or research or evaluation methods without providing findings supported through sound research or evaluation will not be of primary interest to the journal. In addition, submissions that focus solely on describing partnerships, collaborations, and coalition-building efforts will not be of interest to the journal.

## Revisiting Article Types

Moving forward, *PCD* will strongly encourage the submission of manuscripts that align with the journal’s revised mission and areas of interest. Doing so will put the journal’s editorial resources to best use. Given that *PCD* has not received a meaningful number of book reviews for consideration, the Book Review article type has been eliminated. *PCD* also will eliminate the Special Topics and Community Case Study article types. In their place, *PCD* will introduce a new article type, Program Evaluation Brief. Program Evaluation Briefs will allow authors to share promising preliminary data and findings based on the use of sound evaluation methods and approaches. An important goal of this new article type is to encourage more submissions from organizations and institutions (eg, state and local health departments, community-based organizations) with findings from well-delivered and evaluated public health programs. *PCD* also has renamed the Tools and Techniques article type to Tools for Public Health Practice. *PCD* will keep the following article types: Original Research, Research Brief, Systematic Review, Implementation Evaluation, Essay, and Letter to the Editor. *PCD* will place greater emphasis on publishing Program Evaluation Brief, Implementation Evaluation, Original Research, Research Brief, and Systematic Review article types. *PCD* will maintain the highest ethical standards in scientific publishing to promote a transparent review and decision-making process for all manuscripts submitted to the journal.

## Securing Scientific and Programmatic Expertise

To emphasize *PCD*’s commitment to publishing quality articles from around the world, the journal will add the following statement to the journal’s About the Journal statement: “*Preventing Chronic Disease* (*PCD*) is a peer-reviewed public health journal sponsored by the Centers for Disease Control and Prevention and authored by experts worldwide.” Although supported by CDC, the journal maintains its commitment to a broad representation of public health professionals on its editorial board; of *PCD*’s 23 editorial board members, only one is a CDC employee. Over the past year, *PCD* has increased the number of associate editors to improve access to specific content areas. *PCD* currently has 16 associate editors, 9 who are external to CDC and 7 who are CDC employees. The names, titles, affiliations, backgrounds, and appointment terms are available on the journal’s website (https://www.cdc.gov/pcd/about_the_journal/associate_editors.htm). Term limits for all editorial board members and associate editors were established in 2016. We continue to identify new talent and improve succession planning to ensure the journal secures and maintains the necessary expertise.

## Complementing Our Work on Epidemiological Studies With Increased Attention to Evaluating Population-Based Interventions and Policies

*PCD* has developed an international reputation as an authoritative resource, publishing the latest information on the epidemiological effects of behavioral, psychological, genetic, environmental, biological, and social factors that influence health. *PCD* will continue to serve as primary resource to the world in this area, and we will now increase our focus on disseminating articles that report findings beyond epidemiological studies. *PCD* will emphasize identifying and securing articles from researchers and practitioners working in settings that improve health by using population-based interventions and policies. Researchers and practitioners are encouraged to submit Original Research, Implementation Evaluation, and Program Evaluation Brief articles to the journal for consideration.

## Providing Transparent Information on the Journal’s Impact

*PCD* will provide transparent information on its website that describes and reports measures used by the journal to determine the quality of the journal’s content and the journal’s global reach. Multiple metrics will be posted in the journal’s annual *Year in Review* to provide readers with a sense of the journal’s relevance, resonance, reach, routine, and recognition:

1. Relevance: Measure of publication impact by examining the number of *PCD* citations.
2. Resonance: Measures of sharing activity that generates attention to create awareness and dissemination. This will include “likes,” bookmarks, and media coverage (Altmetric).
3. Reach: Measures that help examine how far information can travel to help determine popularity, affinity, and potential impact. *PCD* will track the number of views, downloads, and visitors to its website, and for all published articles.
4. Routine: Measures that provide insight on processes that ensure publication content aligns with the journal’s mission and scope and with the highest publication standards. *PCD* will report on the number of submissions, rejection rate, acceptance rate, and turnaround times in its annual *Year in Review*.
5. Recognition: Measures that rely on citation history to determine the journal’s standing in scholarly literature using various systematic approaches. *PCD* will use impact factor, Scopus, and Google Scholar to help determine the journal’s recognition.

## Conclusion

*PCD*’s commitment to scientific quality and integrity, service to the public, and technological innovation over the past 13 years have led to its respected place in the field of public health. The external review panel was an initiative to continue this success and advance the journal. NCCDPHP’s leadership, along with *PCD*’s editorial board, associate editors, and staff remain committed to enhancing the journal’s focus, reach, and visibility. Its revised vision, mission statement, and areas of focus better emphasize the journal’s commitment to advancing the intersection of research, practice, and policy.

## Appendix *Preventing Chronic Disease* External Review Panel

### Hector Balcazar, PhD, MS (Chair)

Hector Balcazar is dean of the College of Science and Health at Charles R. Drew University of Medicine and Science. Hector specializes in the study of public health problems of Latinos/Mexican Americans. He has conducted numerous studies of Latino birth outcomes, acculturation and health-related behaviors, cardiovascular disease prevention programs in Latinos, and border health issues. He served as an editorial board member for several journals, and served as chair of the editorial board of the *American Journal of Public Health*.

### Ana F. Abraido-Lanza, PhD (Co-Chair)

Ana Abraido-Lanza is professor of sociomedicine and director of the doctor of public health program at the Mailman School of Public Health at Columbia University. In addition, Ana serves as the director of the Initiative for Minority Student Development. Ana’s research focuses on cultural, psychological, and socioeconomic processes that affect psychological well-being, adjustment to chronic illness, and mortality among Latinos. Ana is an associate editor at the journal *Health Education and Behavior* and a member of the editorial board at the *Annals of Behavioral Medicine*.

### Collins O. Airhihenbuwa, PhD, MPH

Collins Airhihenbuwa is former professor and dean of the College for Public Health and Social Justice at Saint Louis University (SLU). He was also the director of the Global Health Institute at SLU. Collins is an internationally recognized expert on behavioral health and a pioneer in centralizing culture in health behaviors. Collins has served on editorial boards for several peer-reviewed journals: *American Journal of Health Behavior*; *AIDS Education and Prevention*; *Health Education and Behavior*; *Journal of Health Communication*; and* Journal of Medical Anthropology*.

### Melissa Grim, PhD

Melissa Grim serves as chair and professor at Radford University in the Department of Health and Human Performance. Melissa’s expertise and interests include planning and evaluating public health interventions to increase physical activity and investigating differences in health behavior in urban, rural, and suburban settings. Melissa currently serves as the deputy editor for *Health Promotion Practice*, a journal dedicated to linking research and practice.

### Shiriki Kumanyika, PhD, MPH

Shiriki Kumanyika is a research professor in the Dornsife School of Public Health at Drexel University. Shiriki retains an appointment as an emeritus professor of epidemiology at the University of Pennsylvania. She was vice-chair of the US Department of Health and Human Services’ Secretary’s Advisory Committee on Healthy People 2020 objectives, is a past president of the American Public Health Association, and is a member of the National Academy of Medicine (formerly known as the Institute of Medicine). Shriki is currently a member of the CDC Task Force on Community Preventive Services, co-chair of the Policy and Prevention Section of the World Obesity Federation, and a member of the Lancet Commission on Obesity.

### William L. Lanier Jr, MD

William L. Lanier is professor of anesthesiology at the Mayo Clinic. His research interests involve neurosurgical anesthesiology and ischemic brain disease, and he has been engaged in both laboratory and clinical research. He was a founding section editor of *Journal of Neurosurgical Anesthesiology* and founding editorial board member for *Disaster Medicine and Public Health Preparedness*. William served as a faculty member for the Council of Science Editors (CSE) for its 2-day Short Course for Editors and director of the CSE’s Short Course. He served as editor in chief of *Mayo Clinic Proceedings*, the world’s third-largest circulation scholarly medical journal. He is emeritus editor in chief of *Mayo Clinic Proceedings.*

### Sarah Patrick, PhD, MPH

Sarah Patrick is director of the Jackson County (Illinois) Department of Health. She directs and manages 3 major divisions of the Department of Public Health. These are Communicable Disease Control, Health Promotion and Public Health Research, and Environmental Health. Sarah has extensive experience developing and supporting collaborative public health practice partnerships between local health departments, academic institutions, and hospital systems. In 2015, she served on a project led by the Council of State and Territorial Epidemiologists to identify scientific writing needs among applied epidemiologists.

